# Engineering Metal-Organic-Framework (MOF)-Based Membranes for Gas and Liquid Separation

**DOI:** 10.3390/membranes13050480

**Published:** 2023-04-29

**Authors:** Yutian Duan, Lei Li, Zhiqiang Shen, Jian Cheng, Kewu He

**Affiliations:** 1 College of Electrical Engineering, Zhejiang University, Hangzhou 310027, China; ytduan@zju.edu.cn; 2 SINOPEC Nanjing Research Institute of Chemical Industry Co., Ltd., Nanjing 210048, China; 3 Department of Orthopedics, The First Affiliated Hospital of University of Science and Technology of China (USTC), Division of Life Sciences and Medicine, University of Science and Technology, Hefei 230001, China; 4 Imaging Center, Third Affiliated Hospital of Anhui Medical University, Hefei 230031, China

**Keywords:** metal organic framework (MOF), MOF-based membranes, gas separation, liquid separation

## Abstract

Separation is one of the most energy-intensive processes in the chemical industry, and membrane-based separation technology contributes significantly to energy conservation and emission reduction. Additionally, metal-organic framework (MOF) materials have been widely investigated and have been found to have enormous potential in membrane separation due to their uniform pore size and high designability. Notably, pure MOF films and MOF mixed matrix membranes (MMMs) are the core of the “next generation” MOF materials. However, there are some tough issues with MOF-based membranes that affect separation performance. For pure MOF membranes, problems such as framework flexibility, defects, and grain orientation need to be addressed. Meanwhile, there still exist bottlenecks for MMMs such as MOF aggregation, plasticization and aging of the polymer matrix, poor interface compatibility, etc. Herein, corresponding methods are introduced to solve these problems, including inhibiting framework flexibility, regulating synthesis conditions, and enhancing the interaction between MOF and substrate. A series of high-quality MOF-based membranes have been obtained based on these techniques. Overall, these membranes revealed desired separation performance in both gas separation (e.g., CO_2_, H_2_, and olefin/paraffin) and liquid separation (e.g., water purification, organic solvent nanofiltration, and chiral separation).

## 1. Introduction

Separation of chemical mixtures is the most energy-consuming process in the chemical industry [1,2]. Traditional separation methods, such as thermally driven phase change separation processes including distillation and regeneration of adsorbents after adsorption and separation [3], consume enormous resources, and the energy consumption of distillation accounts for about 50% of total industrial separation [4]. However, membrane separation technology conducted through molecular size and chemical affinity, not limited to thermal-driven force and adsorbent, has the advantages of energy saving, environmental protection, small floor space, recovery of valuable chemicals, and so on [5,6,7,8,9]. Thus, membrane separation has improved energy efficiency, making it a promising alternative to traditional separation technology [7]. For example, replacing distillation with membrane separation technology in the petrochemical field could save 80% of energy [10] and reduce global energy consumption by 8%. In particular, the membranes consist of inorganic, polymer, and mixed matrix membranes, of which the inorganic membrane has been successfully applied to the separation of CO_2_ and CH_4_ [11]. However, the inorganic membrane is difficult to realize in large-scale industrial applications due to its high capital cost. Meanwhile, although the polymeric membrane is easy to process at a low cost, there still exist problems such as aging, poor thermal stability, and mechanical strength, such as easy plasticization under high pressure during separation and purification. In addition, it is worth noting that there is also an inherent trade-off between the selectivity and permeability of polymer membranes [12], viz., increasing membrane permeability may decrease selectivity, and vice versa.

Later, researchers proposed the concept of metal-organic framework (MOF), which is a class of porous crystalline materials formed by the self-assembly of metal ions (or metal clusters) and organic ligands through covalent and coordination bonds [13]. Compared with commonly used microporous materials for separation, such as activated carbon and zeolite, MOF has a high specific surface area (i.e., 500–7000 m^2^/g) with good thermal/physical stability [14], and the structure, pore size, and functions of MOF could be delicately tailored by metal ions, organic ligands, etc. [15,16,17], thus it is widely used for gas storage, adsorption separation, chemical sensing, catalysis, biological applications, and so on [17,18,19,20]. As early as 2016, MOF adsorbents were used for the storage and subsequent release of 1-methylcyclopropene (1-MCP) to extend the shelf life of fruits and vegetables [21]. MOF membrane separation is one of the most effective methods to maximize the utilization of the potential of MOF-based materials [22], which is expected to serve as a substitute to solve many thorny problems (e.g., stability problems of polymers) for membrane separation.

**Figure 1:**
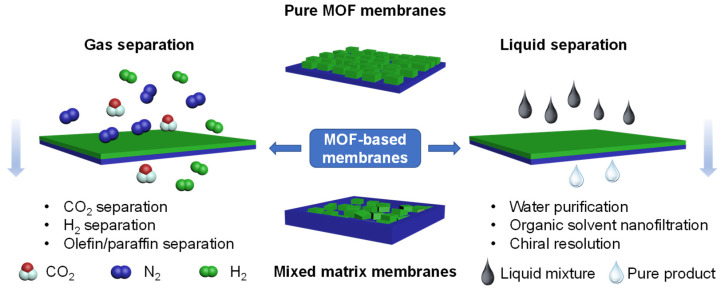
Schematic illustration of extensive applications of pure MOF membranes and MMMs for gas and liquid separation.

MOF-based composites include pure MOF membranes and MOF-based mixed matrix membranes (MMMs), both of which are considered the next generation of MOF materials [4]. To date, numerous high-performance pure MOF membranes and MMMs have been prepared for gas separation (CO_2_, H_2_, olefin/paraffin, etc.) and liquid separation (water purification, organic solvent separation, chiral resolution, etc.) (Figure 1), according to the difference in molecular size and chemical affinity, while the sorption-diffusion model has been employed to describe the permeation phenomenon [23,24]. Herein, a pure MOF membrane is a kind of polycrystalline material assembled from porous substrates (e.g., α-alumina and silica), the separation performance of which mainly depends on framework flexibility [25] and grain boundary structure [26,27], etc. [28]. As another important membrane, MMMs are composed of selective MOF fillers and polymer matrix, combining the excellent transfer characteristics of MOF with the advantages of facile processability and low cost of polymers. Note that the separation performance is mainly affected by the dispersity of MOF fillers, plasticization and aging of the polymer matrix, and interfacial compatibility between them [4,29].

In this review, we highlight the factors affecting the separation performance of MOF-based membranes (i.e., pure MOF membranes and MMMs) and the corresponding solutions, as well as their applications in gas and liquid separation. Herein, the factors affecting the separation performance of pure MOF membranes and MMMs are first described in detail, such as framework flexibility, defect and grain orientation of pure MOF membranes, MOF filler aggregation, plasticization and aging of the polymer matrix, interfacial compatibility of MMMs, and so on. Meanwhile, we describe the application of MOF-based membranes in gas separation, including CO_2_, H_2_, and olefin/alkane separation. Moreover, applications of MOF-based membranes in liquid phase separation are comprehensively reviewed, such as water purification, organic solvent separation, and chiral resolution. Overall, we provide broad insight into this very important topic and look forward to the future of MOF-based membranes as promising functional materials.

## 2. Rational Design of MOF-Based Membranes

Pure MOF membranes and MMMs are the next generation of MOF materials, which could be used for gas and liquid separation as an energy-saving method [30], in place of conventional energy-consuming techniques such as distillation. As a widely known burgeoning material, pure MOF membranes are typically assembled on a porous substrate (e.g., α-alumina and silica), which nevertheless still has restrictive issues of framework flexibility [25], defects [26], orientation [27], and so on [4]. As another important membrane, MMMs have a two-phase composition by incorporating MOF as fillers into the polymer matrix; however, there are problems such as MOF dispersity [31], polymer plasticization, aging [32], interfacial compatibility [33], and so on [34]. Therefore, the composition of MOF and matrix and their compatibility with each other are crucial for their function and efficiency [21]. Herein, the reasonable selection of MOF, substrate, and method of enhancing their interaction will be elaborated in detail in order to guide the preparation of ideal pure MOF membranes and MMMs for gas/liquid separation.

### 2.1. Properties of MOFs

**Figure 2:**
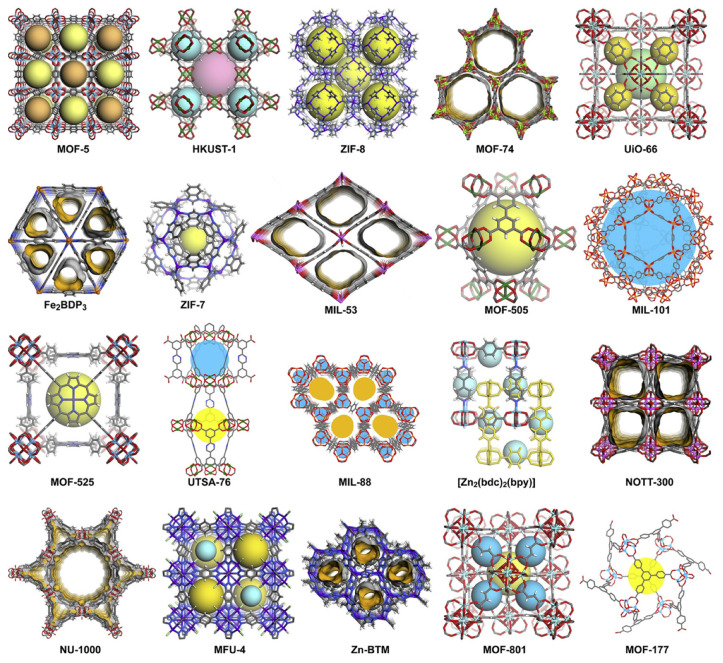
Schematic illustration of the structure of common MOF materials. Reproduced with permission from [14], copyright 2020, Elsevier.

MOF is normally composed of metal nodes and organic linkers. Compared to conventional materials such as silica gel and zeolites, MOFs have outstanding water absorption capacity, recyclability, ease of reconstruction, and unique heat transfer properties [35]. Typical structures of MOF [14] used for membrane preparation are shown in Figure 2, including zeolitic imidazolate frameworks (ZIFs), UiO-66 (i.e., Zr_6_O_4_(OH)_4_(benzene-1,4-dicarboxylato)_6_), HKUST-1 (i.e., Cu_3_(1,3,5-benzenetricarboxylate)_2_), MIL-53, and MIL-101. Due to the diversity of metal nodes and organic linkers in MOF, ~70,000 MOF materials have been synthesized in the past 10 years [36], and the pore size, structure (e.g., slit, tubular, spherical, cylindrical, etc.) [37], and functions formed are multifarious [38]. Therefore, gas or liquid molecules could be separated according to the difference in size and adsorption affinity [39,40]. Note that the size of the MOF could be precisely tailored by isoreticular approaches, framework interpenetration, pore space partition, and other methods. For example, Eddaoudi et al. [41] used an organic linker with different lengths to replace the original MOF linker, wherein the replaced organic linker has the same connectivity and geometry. Herein, the framework structure remained unchanged, but the aperture changed from 3.8 Å to 23.8 Å. In addition to changing the organic linker, the isoreticular approach could also replace metal ions. For example, Shekhah et al. [42] replaced the metal ion of SIFSIX-3-M MOF from Zn to Cu, with the pore size changing from 3.84 Å to 3.50 Å. However, the interaction between the pore surface and molecules could be enhanced with the decrease in pore size; therefore, small molecules could also pass through pore voids and thus be sieved. The improvement of MOF separation performance could be achieved not only through the precise control of apertures but also through the functionalization of MOF. Herein, functionalization could render MOF selectively recognize gas or liquid molecules by selectively enhancing binding affinity between them. Functionalized sites include organic linkers [43] and unsaturated metal sites (i.e., open metal sites, OMSs) [44], of which organic linkers sites could further introduce polar groups (e.g., –NH_2_, –NO_2_, –OH, –Br, and so on) and realize the selective sieving of MOF by intermolecular interactions, such as H-binding.

### 2.2. Pure MOF Membranes

Pure MOF membranes are prepared on porous substrates by some commonly used preparation methods, including in situ growth, seeded growth, layer-by-layer (LbL) techniques, and vapor phase growth [45]. The main factors affecting the separation performance of pure MOF membranes are mainly composed of framework flexibility and grain boundary structure [46], wherein the framework flexibility allows larger molecules to pass through [25], while the poor grain boundary structure could produce defects acting as a nonselective pore to affect the separation performance of membranes [26]. Note that recent reports have demonstrated that defects can help enhance the separation performance of pure MOF membranes rather than cause side effects through defect engineering due to increased porosity and OMSs [47]. In addition, since pure MOF membranes are polycrystalline materials, grain orientation can also affect the separation performance of the membranes [48].

#### 2.2.1. Framework Flexibility

Framework flexibility is the unique nature of MOF, which could increase the effective aperture of ZIF-8 from the original crystallographic pore size of 3.4 Å to 4.0–4.2 Å, thus contributing to C_3_H_6_/C_3_H_8_ separation [4]. However, due to the flexibility of the framework, molecules larger than the pore size may also pass through, thus compromising the separation performance of MOF. For example, Choi et al. [49] prepared the rigid ZIF-8/GNR/alumina membrane by introducing graphene nanoribbons (GNRs) into the ZIF-8 MOF layer. Through strong chemisorption between the sp^2^ carbon edge of GNRs and Zn ions, the ZIF-8 framework generated a strong anchoring effect, inhibiting the framework’s flexibility. The intrinsic aperture size of the membrane is 3.4 Å, which endows it with good H_2_/CO_2_ separation performance. Meanwhile, the membrane has ideal H_2_ permeability (i.e., 5.2 × 10^−6^ mol m^−2^ s^−1^ Pa^−1^) and ideal selectivity (i.e., 142). Another way to suppress the framework’s flexibility is to introduce large ligands. For example, Hou et al. [50] prepared a stiffened ZIF-7_x_-8 membrane by adding benzimidazole linkers with large spatial volumes to ZIF-8_Cm through the mixed-linker strategy and the fast current-driven synthesis (FCDS) method. Considering that the linker mobility is inhibited by the stiff CM phase in ZIF-7_x_-8 and that the aperture is reduced by benzimidazole linkers, the CO_2_/CH_4_ selectivity of the ZIF-7_22_-8 membrane was improved. The separation factor is more than 25, which is about 10 times that of the parent unmodified ZIF-8 film. Note that the aperture of ZIF-8 is 3.8 Å, which is between the kinetic diameters of CO_2_ (3.3 Å) and CH_4_ (3.8 Å), thus avoiding the passage of the CH_4_ molecule due to the flexibility of linkers reported previously [51].

#### 2.2.2. Defect

The dominant reasons for the defect in pure MOF membranes include synthesis conditions and weak membrane-substrate binding affinity [4]. MOF synthesis could produce defects in various processes, such as the cooling process after MOF synthesis at high temperatures, due to the thermal expansion coefficient of the substrate, which is difficult to match with MOF, which has large negative thermal expansion coefficients [40]. Herein, extending the cooling period after MOF synthesis at high temperatures is conducive to reducing related defects. When exposed to environmental impurities such as water vapor, H_2_S, and NO_x_, MOF is liable to degradation caused by water vapor, and the combination of H_2_S and NO_x_ with OMSs could block absorption sites, thus damaging MOF stability, where defects are prone to occur and further compromise MOF performance [52]. Therefore, MOF needs to be functionalized for modification (e.g., de novo synthesis, postsynthetic modification, one-step bottom-up approach, etc.) to improve stability and tolerance under harsh conditions such as exposure to moisture/water, and acid/base. For example, Zhu et al. [53] transformed the hydrophilic MOF UiO-67 into hydrophilic UiO-67-Rs (R = alkyl) by introducing alkyl chains into organic linkers. The UiO-67-Rs exhibited prominent acid/base and water stability; such MOFs have promising prospects for water purification.

The MOF membrane could produce detachments and then form non-selective channels when there are poor interactions between MOF and substrate, which compromise the membrane separation performance. Chemical modification or physical interlocking (e.g., “counter-diffusion” or “interfacial growth” methods) are performed on the MOF and substrate to enhance interactive adhesion. Chemical modifications utilize some compounds (e.g., 3-aminopropyltriethoxysilane (APTES), PDA, and 2-methylimidazole) as linkers to functionalize the substrate or MOF and form interactions between them (e.g., coordination, covalent interaction). For example, Liu et al. [54] modified the porous α-Al_2_O_3_ matrix tube covalently with APTES to prepare a high-quality ZIF-9 membrane, wherein the amino group of APTES could coordinate with Zn^2+^ as a nucleation site to promote the generation of continuous ZIF membranes and improve membrane separation performance. The mixture separation factors of H_2_/CO_2_, H_2_/CH_4_, and H_2_/N_2_ in the ZIF-9 membrane were 21.5, 8.2, and 14.7, respectively, which far exceeded the corresponding Knudsen coefficients. The membrane also exhibited better stability and promising potential for H_2_ industrial separation. In addition to strengthening the bond between MOF and the substrate through functional modification, the substrate (i.e., metal net) can also be directly used as an additional metal source for MOF preparation. For example, Guo et al. [55] used Cu net as the substrate and PI-CuBTC (PI = polyimide; CuBTC = Cu-benzene tricarboxylate) suspension as the sealing agent to prepare high-quality ZIF-8/PI-CuBTC (50%) membranes with H_2_/CH_4_ selectivity of 71.0. Therein, Cu^2+^ ions in the Cu net could form a Cu-N interlayer with 2-methylimidazole, which then acted as nucleation sites to promote the growth of the ZIF membrane.

However, recent defect engineering introduced defects into MOF crystalline materials, and the MOF separation performance was not reduced but unexpectedly improved. The reason behind this was the increase in porosity and OMSs. Wang et al. [56] prepared the highly defective MIL-125 membrane using the Ti_8_(μ_2_-O)_8_(OOCC_6_H_5_)_16_ cluster as a Ti source by combining single-mode microwave heating with tertiary growth. The gas separation performance of the membrane was improved by controlling the number of missing-linker defects, with the ideal selectivity of CO_2_/N_2_, H_2_/N_2,_ and H_2_/CH_4_ up to 38.7, 64.9, and 40.7, respectively. Note that the selectivity of CO_2_/N_2_ was higher than the technical index of existing pure MOF membranes under similar conditions.

#### 2.2.3. Orientation

**Figure 3:**
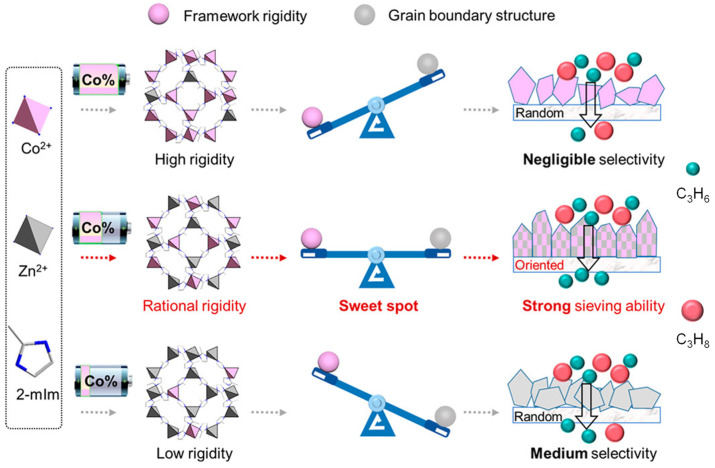
Schematic illustration of the design of bimetallic Zn_(100−x)_Co_x_-ZIF MOF membranes. Reproduced with permission from [58], copyright 2020, American Chemical Society.

The orientation control of the polycrystalline MOF is effective in improving the grain boundary structure [27]. When the crystals of the MOF membrane grow perpendicular to the substrate surface to form the oriented MOF membrane, the grain boundary defect can be reduced, thereby improving the separation performance. Ma et al. [57] prepared *c*-oriented ZIF-95 membranes through the vapor-assisted in-plane epitaxial growth method. Compared with the corresponding randomly oriented ones, the *c*-oriented ZIF-95 membranes had fewer intercrystalline defects and transport paths, thus greatly improving the separation ability. The H_2_ permeance of *c*-oriented ZIF-95 membranes (over 7.9 × 10^−7^ mol m^−2^ s^−1^ Pa^−1^) is 4.6 times that of the corresponding randomly oriented ones, and the mixture separation factors of H_2_/CO_2_ and H_2_/CH_4_ increased to 32.2 and 53.7, respectively. In addition, a rigidity factor could be introduced to preferred orientation membranes, which can improve separation performance by balancing grain boundary structure and the rigidity of the framework. Hou et al. [58] prepared a crystal-oriented Co-Zn bimetallic ZIF membrane (Zn_82_Co_18_-ZIF) by the FCDS method, wherein Co^2+^ worked as the rigidity factor to influence the framework flexibility (Figure 3). The resulting bimetallic membrane has a perfect balance between framework rigidity and grain boundary structure, and meanwhile, the membrane separation factor of C_3_H_6_/C_3_H_8_ is as high as 200.

### 2.3. Mixed-Matrix Membranes

The MMM is composed of MOF particle filler and polymer substrate, wherein the commonly used polymer substrate includes poly(methyl methacrylate) (PMMA), PI, poly(vinylidene fluoride) (PVDF), polysulfone(PSF), polyethersulfone (PES), etc. [34]. Due to the fact that permeance and selectivity are inversely correlated with highly permeable membranes lacking selectivity and vice versa, the separation performance of polymeric membranes is limited, which could be improved to exceed the Robeson upper bound limits by adding MOF particles to the polymer matrix. However, MMM’s capability for gas and liquid separation is dented due to the aggregation of MOF particles [31], the plasticization and aging of polymer [32], and the interfacial compatibility [33] between MOF and matrix. Therefore, first and foremost, the aforementioned concerns need to be properly addressed in order to prepare high-performance MMMs.

#### 2.3.1. MOF Particle Aggregation

Macrovoids caused by MOF particle aggregation locate the MMM interface of MOF and polymer, forming non-selective channels and further compromising the separation performance of the membrane [59]. MOF particles of high load and large size are prone to aggregation; due to that, the interaction between MOF and MOF is greater than that between MOF and polymer. Here, the MOF load of most MMMs is not high. However, if MOF of high content could be uniformly distributed by enhancing interaction with the polymer matrix, MMM selectivity could be reinforced. For example, He et al. [60] prepared the defect-free ZIF-8/polymers of intrinsic microporosity (PIM-1) MMM with ultra-high ZIF-8 loading capacity through a symbiosis-inspired de novo strategy. Since the CN group on the PIM-1 matrix could interact with the uncoordinated imidazole groups (NH groups) on ZIF-8, the interface compatibility is accordingly enhanced. Note that the MOF was still uniformly distributed despite the increased loading capacity of 67.2 wt%, and meanwhile, the CO_2_/N_2_ selectivity of this MMM was increased to 24.4 ± 2.3, with a maximum CO_2_ permeability of 6338 Barrer. Furthermore, this method was also applicable to ZIF-7/PIM-1 and ZIF-67/PIM-1 MMMs with high loading capacities of ZIF-7 (i.e., 71.9 wt%) and ZIF-67 (i.e., 82.5 wt%), and H_2_/N_2_ and CO_2_/N_2_ selectivity of 0.1-ZIF-67/PIM-1 could reach up to 24.5 and 34.5, respectively. In addition, small MOF particles prepared by controlling nucleation and growth rate were added to the polymer matrix, and the prepared MMM presented the desired particle dispersion and interface compatibility, thus improving the separation performance. For example, Xiong et al. [61] replaced the original ligand 2-methylimidazole (2-MeIM) with 2-aminobenzimidazole (2-amBzIM) to prepare mixed-linker ZIF particles with smaller particle sizes (i.e., mixed-linker ZIF-8-NH_2_ particle size: ~30–40 nm, parent ZIF-8 particle size: ~50–60 nm). ZIF-8-NH_2_/poly(vinyl alcohol) (PVA) MMMs were prepared by doping ZIF-8-NH_2_ particles as filler into PVA, which had good particle dispersity, interface compatibility, and hydrophilicity. In addition, the membrane exhibited good water/ethanol separation performance, wherein the water/ethanol separation factor could reach up to 925 with a comparable total flux of 142 g m^−2^ h^−1^, which was 524% and 73% higher than the counterpart of parent ZIF-8/PVA MMMs, respectively.

#### 2.3.2. Plasticization and Aging of Polymer Matrix

The MMMs could cause the reorganization of macromolecular chains when used for the separation of condensable and polarizable gases, thus leading to polymer plasticization. Note that plasticization could increase the spacing and mobility of polymer chains, making the membrane gradually lose gas selectivity. Through the use of MOF containing unsaturated metal sites [62] or flexible two dimensional MOF (2D MOF) (e.g., Cu(dhbc)_2_(bpy)·H_2_O) [63] and ionic liquid, the interaction between MOF and polymer matrix could be enhanced, thus reducing the polymer chain mobility and forming anti-plasticizing MMMs with desired CO_2_ separation performance. In addition, Wang et al. [63] used MOF covalently grafted with PI brushes as noncovalent cross-linking nodes. Through strong brush-brush interaction, polymer chains are connected to the surrounding ones, which limited the mobility and enhanced the plasticizing resistance of MMM and simultaneously endowed the membrane with ideal CO_2_/CH_4_ and CO_2_/N_2_ separation capabilities.

The matrix polymers reached thermodynamic equilibrium over time by rearranging their spatial positions. This aging process could release the free volume trapped along the polymer chains and tighten the polymer chain packing, which greatly reduced MMM gas permeability. Note that this aging usually occurs on rigid polymer or PIM-based membranes. Generally, the aging rate of MMM could be delayed by MOF crosslinking with the polymer matrix. For example, Nguyen et al. [64] constructed a cross-linked structure between UiO-66-NH_2_ and PIM-1, which mitigated the physical aging behavior, and the membrane permeability was still 80% of the initial value even after 400 days.

#### 2.3.3. Interfacial Compatibility

The interfacial compatibility between the MOF phase and the polymer phase in MMM is the main factor affecting membrane separation performance. Poor compatibility will result in the formation of non-selective interfacial voids, polymer rigidification, pore clogging, etc. [65], thus reducing the separation performance of the membrane. Interfacial compatibility can be enhanced by using similar MOF and matrix properties. For example, Chen et al. [66] prepared UTSA-280/6FDA-polyimide MMMs by mixing UTSA-280 bearing C_2_H_4_/C_2_H_6_ molecular sieving functions as filler with a 6FDA-DAM:DABA (3:2) polymer matrix with matched low permeability. The prepared MMMs exhibited homogenous dispersion and satisfactory interfacial compatibility. The C_2_H_4_/C_2_H_6_ separation performance of the obtained MMMs was significantly improved with optimized C_2_H_4_ permeability of 6.49 Barrer and C_2_H_4_/C_2_H_6_ selectivity of 4.94. Further, the C_2_H_4_ permeance and C_2_H_4_/C_2_H_6_ selectivity of UTSA-280/6FDA-polyimide MMMs were increased by 15% and 32%, respectively, compared to MMMs prepared from 6FDA-DAM polymeric matrix with poor permeability. However, given the large variety of MOF materials, it is difficult to find appropriate polymer matrices with similar properties. Herein, a large number of methods have been developed to enhance interfacial compatibility, including chemical modification, regulation, and control of MOF topological structures, OMS coordination, derivative interaction, hydrogen bonding, and secondary interfacial polymerization. For example, He et al. [67] adjusted the MOF aperture through the double solvent approach by dopamine polymerization of [Ni_8_(L)_6_]n (Ni-MOF). Due to the hydrogen bonds formed between RNH_2_/R_2_NH groups in polydopamine and R_3_N groups on polymer, the interfacial compatibility between MOF and 6FDA-DAM polymer matrix was enhanced, reducing the interface defects of Ni-MOF/PDA/6FDA-DAM MMMs. The C_3_H_6_/C_3_H_8_ separation performance was therefore improved compared to pure polymer; the C_3_H_6_/C_3_H_8_ separation performance of the MMM was improved with superior propylene permeability (i.e., ~90 Barrer) and propylene/propane selectivity (i.e., ~75).

**Figure 4:**
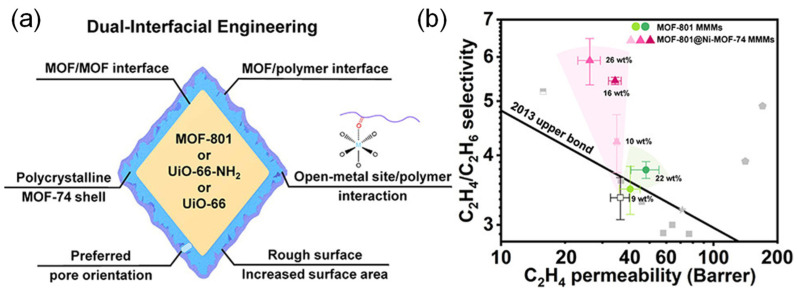
(**a**) Schematic illustration of the MOF-801@Ni-MOF-74 MMMs, UiO-66-NH_2_@Ni-MOF-74 MMMs, and UiO-66@Ni-MOF-74 MMMs were prepared by dual-interfacial engineering method. (**b**) The separation performance of MOF-801@Ni-MOF-74 MMMs. Reproduced with permission from [68], copyright 2020, American Chemical Society.

In addition to directly enhancing the interaction between filler and matrix, a dual-interfacial engineering approach can also be used to form MMM containing both MOF-MOF and MOF-polymer interfaces, thus enhancing the compatibility between MOF and polymer. Wu et al. [68] prepared MOF-801@Ni-MOF-74 MMMs through a dual-interfacial engineering approach by adding MOF-74 with high-density open metal sites to the layer between MOF-801 and the polymer matrix (Figure 4). Note that the resulting product contained two interfaces, namely, MOF-MOF and MOF-polymer interfaces. Moreover, the interfacial compatibility of MOF and polymer was improved by complementary interactions between open metal sites of MOF-74 and hydroxyl groups. Meanwhile, microwave-assisted lattice matching between MOFs facilitated the vertically oriented growth of Ni-MOF-74 crystallites and further inhibited the horizontal transport of gas molecules. The aforementioned factors significantly improved the selectivity of ethylene/ethane separation, which is up to 5.91. Similarly, the MOF-74 shell could be applied to UiO-66-NH_2_ to prepare MMMs, which could improve the CO_2_/CH_4_ selectivity and CO_2_ permeance of UiO-66-NH_2_ MMMs simultaneously, similar to the application to MOF-801. Additionally, the ionic liquid layer could also facilitate the combination of the two phases of MOF and polymer, which had a function similar to MOF-74, thus improving the separation performance of MMMs [69].

The high-performance MMMs produced by the above methods are defect-free. Just as defective pure MOF membranes with ideal separation performance could be obtained by defect engineering, so can defective MMMs [70]. Lee et al. [71] used defects as an additional freedom degree to adjust MOF performance and prepared advanced MMMs through the “defect engineering” method. They used trifluoroacetic acid (TFA) as a modifier to prepare defective UiO-66 nanoparticles. Then nanoparticles obtained were used as fillers to prepare defective UiO-66/6FDA-DAM MMMs with 6FDA-DAM polyimide of good C_3_H_6_/C_3_H_8_ separation performance by solution casting method. Since hydrogen bonds were formed between two phases, the membrane has good interfacial compatibility. Meanwhile, the defects of UiO-66 could be used as an additional transport highway for MMMs. However, Zr-C_3_H_6,_ formed by the complex of unsaturated open metal sites and C_3_H_6_, is beneficial for C_3_H_6_ adsorption. Therefore, compared to the pure 6FDA-DAM polymer membrane, the MMM exhibited improved C_3_H_6_/C_3_H_8_ separation performance with enhanced stability, of which C_3_H_6_ permeability could reach 237 Barrer with a C_3_H_6_/C_3_H_8_ selectivity of 9.8.

### 2.4. Properties of MOF Membranes

#### 2.4.1. Stability

Stability is a prerequisite for material performance, and many efforts have been made to improve the stability of MOF-based materials against chemical changes induced by active liquids and/or gases through exquisite structural design and functionalization [4,72]. Notably, ZIFs consisting of imidazolate ligands and metal nodes possess prominent stability due to their zeolite-like structure. The stability of other water-stable MOFs, such as MIL-53 (Al^3+^), UiO-66 (Zr^4+^), MIL-100, and MIL-101 (Cr^3+^), can be enhanced by using highly charged metals to form strong metal-linker bonds, which presented outstanding water stability when used for membrane separation [21]. For example, Hu et al. [73] used the MIL-53 membrane for dehydration of the azeotrope of ethyl acetate (EA) aqueous solution by pervaporation. The MIL-53 membrane maintained high stability during evaporation for over 200 h, and hydroxyl groups on the MIL-53 surface formed hydrogen bonds with water molecules, promoting water transport with a flow rate of 454 g m^−2^ h^−1^. Liu et al. [74] used the UiO-66 membrane for water softening. Note that the Uio-66 membrane could maintain excellent stability and exhibited good multivalent rejection effects lasting ~170 h in different saline solutions (i.e., Ca^2+^, Mg^2+^, and Al^3+^). Among them, the ZIF-8/(TA-Zn^2+^)_2_/PES membrane displayed almost no changes in both pure water permeance and Na_2_SO_4_ rejection after long-term filtration for 100 h, indicating remarkable stability. Moreover, when Jian et al. [75] prepared Al-MOF membranes (two-dimensional (2D) monolayer aluminum tetra-(4-carboxyphenyl) porphyrin framework), no Al^3+^ dissolution was detected after immersion in water for one month, which revealed good water stability as well.

In liquid separation, MOFs are generally stable, although metal ions (e.g., Zn^2+^) could release from the matrix into the surrounding medium [19,76]. Notably, the hydrolysis of MOFs (e.g., ZIF) in aqueous media under ambient conditions was associated with the release of metal ions (e.g., Zn^2+^) that feature a well-established acid sensitivity [19,76]. Over time, the release rate first increases and then decreases, resulting in long-term sustained release [77]. Whereas MOF stability decreases with an accelerated rate of degradation in a weakly acidic environment (e.g., pH = 5.5), wherein metal ions (e.g., Zn^2+^) could be released at a higher rate than in neutral conditions [76,77]. For example, Schnabel et al. [78] prepared the Zn-MOF-74, comprising Zn(II) ions and 2,5-dihydroxybenzene-1,4-dicarboxylate ligand, wherein the detected amount of Zn^2+^ was merely ~0.1% during the whole time period after being tested for 160 h in neutral aqueous conditions (pH = 7.4) and ~0.8% at pH 6.0. Note that with the slow degradation of MOF materials, the long-term release of metal ions could be achieved, which greatly reduces the toxicity of metal ions and prolongs the function time [79,80,81].

Notably, the stability of MOF membranes could be affected by humidity and corrosive and acidic substances when used for gas separation. For example, moisture could cause the MOF OMS to be inactive. Meanwhile, H_2_S, SOx, and NOx can disrupt weak ligand-metal linkages in MOF. In addition, heavy hydrocarbons (such as heptane or toluene) can damage pores or active sites of MOF membranes, thus affecting stability [4]. Herein, the stability of MOFs could be improved by various methods, such as structural changes and functional modifications. For example, when ZIF-8 with a sodalite (SOD) topology is exposed to SO_2_ under high humidity, it is unstable with a damaged structure. However, the stability of ZIF with RHO topology was improved, showing stability for both SO_2_ and CO_2_ even under highly humid conditions [4]. Moreover, MIL-125(Ti)-NH_2_, obtained after amine functionalization of MIL-125(Ti), exhibited high H_2_S stability [82]. Furthermore, the stability of UiO-66 MOF-based MMMs could also be improved through a defect-engineered strategy, where performance remained unchanged even after industrial separation of C_3_H_6_/C_3_H_8_ under harsh conditions (i.e., 50 °C and 5 bar) for 14 days, displaying outstanding stability [71].

#### 2.4.2. Lifetime

Note that MOFs (e.g., ZIF) demonstrated remarkable chemical resistance and thermal stability (e.g., up to 550 °C in N_2_ for ZIF) against solvents because of the strong interactions between core metal ions and ligands [83], which could greatly extend the lifetime of MOF-based materials in both liquid and gas separations for practical applications [27,84]. For example, ZIF-8, which is made up of imidazolate anions forming a tetrahedral joint with ZnN4, has ideal stability (~7 days) in phosphate buffer (pH = 7.4) at 37 °C. Meanwhile, ZIF-8 and ZIF-11 could maintain their crystalline structures in water at 50 °C for 7 days [85]. Liu et al. [86] reported a series of continuous UiO-66 polycrystalline membranes on pre-structured yttria-stabilized zirconia hollow fiber supports. UiO-66 membranes as prepared could maintain ideal separation performance during a 300 h stability test and remain robust even under harsh conditions (e.g., boiling benzene, boiling water, and sulphuric acid). Deng et al. [87] fabricated a novel superwetting HKUST-1 membrane by seed-mediated growth. Notably, the permeation fluxes in both cases (NaCl and pH effect) decreased with increasing time, and the flux was still high at 7 days after washing the membrane immersed in an acid-based solution, which demonstrated the outstanding stability of the HKUST-1 membrane in acidic, basic, and salty environments. Furthermore, several continuous and dense ZIF-8 polycrystalline membranes were fabricated and tested for seawater desalination [88]. The obtained ZIF-8 membranes showed high ion rejection of >99% and excellent water flux of ~6 kg m^−2^ h^−1^ at 25 °C. Notably, these ZIF-8 membranes could maintain good separation performance even during a stability test of up to 7 days.

Furthermore, related studies have shown that MOF membranes also exhibit exceptional robustness and a long-lasting lifetime for gas separation [27,84]. For example, Zhou et al. [89] pioneered the electrochemical synthesis of a series of defect-free face-centered cubic (fcu)-MOF polycrystalline membranes with molecular sieving capacities for hydrocarbon separations, including butane/isobutane (*n*C_4_/*i*C_4_) and C_3_H_6_/C_3_H_8_ mixtures. Thanks to the rigid structure of the framework, the optimized membrane demonstrated outstanding and stable nitrogen rejection performance even after a continuous permeation test over 150 days. Later, they reported the rational design of various MMMs with high loading content (~60 wt%) of (001)-oriented AlFFIVE-1-Ni nanosheets for efficient CO_2_/H_2_S/CH_4_ separation [90]. Notably, the optimized MMM could maintain outstanding separation capacity even after a long-lasting stability test of more than 30 days. This work distinctly highlights the promising potential of utilizing MOF membranes with unprecedented separation abilities. Hou et al. [50] proposed a mixed-linker strategy to prepare a series of ZIF-7_x_-8 hybrid membranes using Zn^2+^ cations and 2-methylimidazole mixtures as ligands via the FCDS approach (x represents the molar percentage of benzimidazole). Moreover, these hybrid membranes could maintain separation performance even after a 180 h temperature swing separation test, consolidating their structural stability and robustness. Moreover, Sabetghadam et al. [91] prepared a MOF/PIMAT membrane, resulting in both a substantial enhancement of CO_2_/N_2_ selectivity and CO_2_ permeability under both dry and humid conditions while greatly reducing aging. The MMM obtained exceeds the 2008 Robeson upper bound limit and reaches the economic target zone for post-combustion CO_2_ capture even after 510 days of aging.

#### 2.4.3. Environmental Friendliness and Biocompatibility

Environmentally friendly and biocompatible MOFs have already played a crucial and prerequisite role in practical applications, including bio-MOFs or biomimetic MOFs [92,93]. Notably, MOFs based on amino acids and their derivatives are widely recognized for their environmental friendliness and biocompatibility. For example, Huang et al. [94] prepared “green” MOFs with aspartic acid derivatives (i.e., spartic acid, succinic acid, fumaric acid, and malic acid) as organic ligands and Zr(IV) as central ions as materials, which are ideal eco-friendly candidates for phosphorus and arsenic(V) removal in complex real water bodies. Moreover, some metals are essential trace metal elements for humans and are not considered toxic for specific applications. For example, the recommended daily calcium intake is ~1 g, which is considered highly biocompatible. Therefore, Ca-MOFs are biocompatible materials with environment-friendly properties that are green, safe, and sustainable [95]. Overall, the factors affecting the biocompatibility of MOFs include coordination metals, organic ligands, particle size, morphology, shape, surface charge, and so on [92]. For coordination metals, the median lethal dose of different metal elements and chemical formulas (counter ion and oxidation states) varies. In particular, organic ligands can be divided into exogenous and endogenous ligands, where exogenous ligands can enhance their biocompatibility through functionalization, while endogenous ligands have essentially good biocompatibility. Therefore, in order to improve MOF compatibility, endogenous metal ions and ligands should be used as much as possible during the synthesis stage. In addition, relevant studies have shown that MOFs have good biocompatibility when particle size is below 200 nm with a hydrophilic surface and stability at physiological pH [92].

#### 2.4.4. Fouling

Notably, fouling refers to the deposition and accumulation of undesired materials on the surface or inside of a membrane (such as dissolved particles, organic macromolecules, inorganic macromolecules, and biological micro-organisms) [96], which presents the greatest challenge to a more widespread use of membrane separation, a potentially energy-efficient and cost-effective separation procedure, in a wide range of industrial sectors [97]. Note that reverse osmosis is currently one of the most successful membrane-based water purification processes in industrial seawater desalination, yet it remains critically affected by membrane fouling [98]. Thus, the pressure would be increased to maintain the high flux, further resulting in an increase in energy requirements [96]. However, Prince et al. developed a self-cleaning PANCMA-PEI-Ag modified PES membrane; (PANCMA = poly(acrylonitrile-*co*-maleic acid); PEI = polyethyleneimine) that afforded a delicate solution to fouling in membrane separation processes. Wherein the flux drop for the novel membrane is lower (16.3% of the initial flux) during long-term experiments with protein solution. Moreover, the novel membrane continues to exhibit inhibition of microbes even after 1320 min of protein filtration, which opens up a promising MOF membrane-based solution for biofouling in wastewater purification [99].

## 3. Gas Separation

Gases are easy to form mixtures that are difficult to separate due to their invisible, high diffusion, and low concentration characteristics [14]. MOF membranes have been widely used for gas separation of CO_2_/N_2_, CO_2_/CH_4_, olefin/paraffin (e.g., C_2_H_6_/C_2_H_4_ and C_3_H_8_/C_3_H_6_), and so on (Table 1), owing to their flexible pore sizes and channels [21]. MOF membranes have remarkable molecular sieving performances for gas separation based on differences in chemical affinity and size between different gas molecules. Note that this method differs from conventional ones such as low-temperature distillation, which is dependent on the difference in boiling point for separation and greatly decreases energy consumption.

### 3.1. CO_2_ Separation

Natural gas is a clean and efficient fuel source for generating large volumes of electricity for power grids, which constitutes an important part of the modern energy system [115]. However, contaminated CO_2_ could reduce the energy content (calorific value) of natural gas, resulting in adverse effects and huge losses [116]. Additionally, CO_2_ exists in hydrocarbon fuel gas, plays the role of a conventional pollutant, and contributes to global warming as one of the best-known greenhouse gases. Therefore, the separation of CO_2_ is fascinating and has caused widespread social concern. Several MOF membranes have hitherto been reported for the separation of CO_2_ from gaseous mixtures, such as CO_2_ (3.3 Å)/CH_4_ (3.8 Å), CO_2_ (3.3 Å)/N_2_ (3.6 Å), and so on.

In CO_2_/CH_4_ separation, Chiou et al. [105] prepared a MOF membrane (CAU-10-H) with a high CO_2_ selectivity due to a favorable coulombic effect using aluminum hydroxide isophthalate MOF. Note that the CAU-10-H membrane is the first case of a pure MOF membrane with CO_2_/CH_4_ selectivity >30, which meets the industrial separation requirements with ideal selectivity of CO_2_/N_2_ (i.e., 42) and CO_2_/CH_4_ (i.e., 95), respectively. However, the CO_2_ permeance reached 500 Barrer, which was higher than the existing index of a pure MOF membrane. In addition to preparing CAU-10 membrane on porous α-alumina disks, Fan et al. [100] combined CAU-10 and MIL-160 membranes through the ligand mixing method to prepare fluorine-functionalized MIL-160/CAU-10-F membrane. Due to the strong affinity of polar fluoro functional groups for CO_2_, the CO_2_/CH_4_ separation selectivity and CO_2_ permeance of MIL-160/CAU-10-F increased by 10.7% (78) and 31.2% (716 GPU) compared to the MIL-160 membrane, respectively. In order to increase CO_2_ separation of MMM by enhancing interactions between two phases, i.e., MOF and polymer matrix, Zhang et al. [101] prepared MMM (PI-IL/MOF) with amino-functionalized UiO-66-NH_2_ MOF and an ionic liquid-capped PI matrix. The interfacial compatibility between UiO-66-NH_2_ MOF and PI matrix was enhanced by the encapsulation effect of ionic liquid; meanwhile, the CO_2_ permeance reached 7.61 Barrer, which should be attributed to the reinforcement effects of UiO-66-NH_2_ as CO_2_ carrier sites. Moreover, the CO_2_/CH_4_ selectivity could achieve the highest value of 95.1, close to the 2008 Robeson upper bound. Here, selectivity and permeance were simultaneously improved.

**Figure 5:**
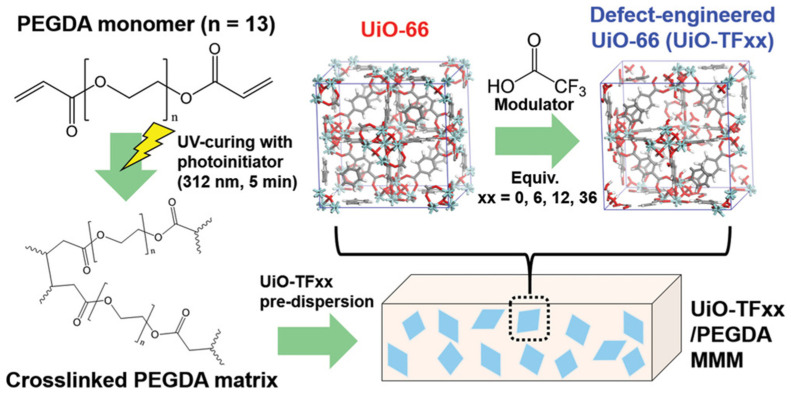
Schematic illustration of the preparation of defect-engineered UiO-66/PEGDA MMMs for CO_2_ separation. Reproduced with permission from [105], copyright 2021, John Wiley and Sons.

For CO_2_/N_2_ separation, Qu et al. [102] added CF_3_COO^-^ with strong electronegativity to ZIF-8 and fixed the rotation of the ligand 2-methylimidazole of ZIF-8 by electrostatic interaction, thus maintaining the four-membered window size at 3.4 Å. Correspondingly, CO_2_/N_2_ selectivity was increased to 137 with a high permeance of 286 Barrer, exceeding the newest 2019 upper bound. In addition to improving the framework rigidity, the strong interfacial interactions greatly promote the separation performance of CO_2_/N_2_. Ashtiani et al. [103] modified UiO-66-NH_2_ MOF covalently with PVA using the “grafting onto” method, which was then combined with the poly(vinyl amine) (PVAm) matrix to afford UiO-66-NH_2_-PVA-PVAm MMM. Note that interfacial compatibility between MOF and PVAm was increased on account of the grafted PVA on MOF, and as CO_2_ carrier sites, amino content increased with the enrichment of UiO-66-NH_2_ MOF in MMM. Wherein CO_2_ is preferentially permeated by the reversible reaction between bicarbonate and amino groups under wet conditions, thereby improving the CO_2_/N_2_ separation performance. Subsequent results demonstrated that the CO_2_/N_2_ selectivity could reach ~45 with a MOF content of 24 wt%. However, Lee et al. [104] first prepared defect-engineered UiO-66 MOF nanoparticles using monocarboxylic acids instead of dicarboxylic acids, then obtained defect-engineered UiO-66/poly(ethylene glycol) diacrylate (PEGDA) MMMs by combining the pre-synthesized MOF with the crosslinked PEGDA matrix (Figure 5). Further results suggested that the MMM had good interfacial compatibility, and defects in UiO-66 hardly affected the interfacial performance between MOF and PEGDA. Moreover, the CO_2_/N_2_ separation performance is significantly improved with the increasing defect content in UiO-66, where the CO_2_/N_2_ selectivity reaches 41 with a CO_2_ permeability of 470 Barrer. This work demonstrated that defects in MOF could be used as a key factor to adjust the CO_2_/N_2_ separation performance of MMM, which was consistent with the simulation results.

### 3.2. H_2_ Separation

As an eco-friendly energy source, hydrogen (H_2_) is emerging as one of the leading options for storing energy from renewable sources and is beneficial to sustainable development [117]. Hydrogen is always produced industrially from natural gas through the water-gas shift reaction after steam reforming of natural gas [118]. However, other by-products, mainly CO_2_, could also be produced concomitantly. Therefore, H_2_/CO_2_ separation is required to purify H_2_. It is noteworthy that the kinetic diameters of H_2_ and CO_2_ differ by 2.89 Å and 3.3 Å, respectively; as a result, H_2_ could be separated by membranes with molecular sieving effects. Meanwhile, other gas mixtures containing hydrogen, such as H_2_/CH_4_ and H_2_/N_2_, could also be separated according to a similar principle.

**Figure 6:**
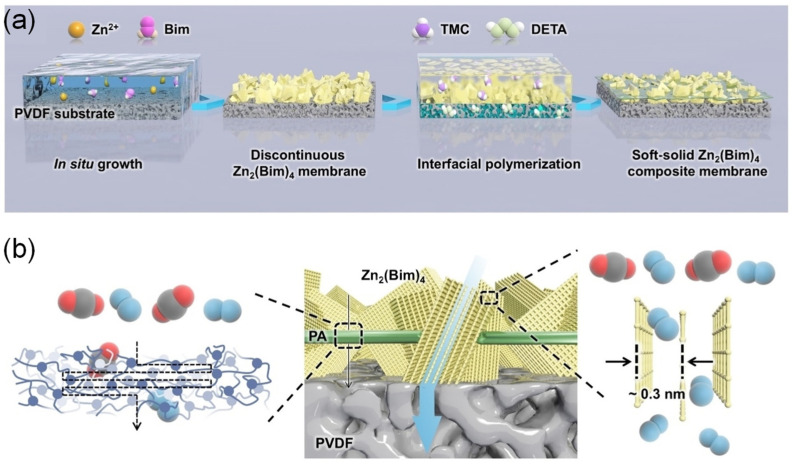
(**a**) Schematic illustration of the preparation of quasi-ab plane preferential-oriented Zn_2_(Bim)_4_ SSCM. (**b**) Schematic illustration of the cross-sectional view of the Zn_2_(Bim)_4_ SSCM. Reproduced with permission from [106], copyright 2022, John Wiley and Sons.

Shu et al. [106] prepared the soft-solid MOF composite membrane (Zn_2_(Bim)_4_ SSCM) with preferentially oriented quasi-*ab* plane for H_2_/CO_2_ separation, using Zn_2_(Bim)_4_ (Bim = benzimidazolate), commercial PVDF substrate, and lateral ultrathin polyamide (PA) film, through a facile strategy combining in situ solvothermal synthesis and confined interfacial polymerization (Figure 6). The Zn_2_(Bim)_4_ SSCM exhibited a remarkable H_2_ permeance of 1313 ± 92 GPU and an unprecedented H_2_/CO_2_ selectivity of 1084 ± 80, which far transcended MOF-based membranes that have been reported. The branched MOF can also be used to prepare MMMs. For example, Lee et al. [107] prepared branched-ZIF-8 (BZ) through a fast-nucleation approach using an amine-based modulator and further prepared BZ MMMs for H_2_ separation using 6FDA-DAM as a polymeric matrix. The branched ZIF-8 (BZ) has high surface-area-to-volume (SA:V) ratios, which could enhance the interaction between MOF and polymers, and the membrane exhibited great separation performance with ideal H_2_ permeability (1528 Barrer) and H_2_/CH_4_ selectivity (19.45). For H_2_/CO_2_ and H_2_/CH_4_ separation, MMMs also exhibited superior separation performance for H_2_/CO_2_, H_2_/N_2_, and H_2_/CH_4_. Li et al. [108] prepared defect-free and roughly oriented hetero-polycrystalline MOF (HPM) hollow fiber membranes for H_2_ separation and purification, using PVDF as hollow fiber substrate through a one-pot synthesis method. Since there was simultaneous hetero-crystallization and hetero-linker coordination during the membrane preparation process, the HPM membranes consisted of Zn_2_(BIM)_4_ MOF phases with a 2D stacking layered structure and ZIF-8 MOF phases with a 3D cubic topology. In addition, there were ultra-small paths in both the 2D lamellar and narrow window of the 3D sodalite phases, and the linker rotation was suppressed simultaneously, resulting in a rigid framework that significantly enhanced H_2_ permanence (over 1100 GPU). The separation selectivity of H_2_/CO_2_, H_2_/N_2_, and H_2_/CH_4_ is very high, which is 361, 482, and 541, respectively.

### 3.3. Olefin/Paraffin Separation

Olefins, also known as alkenes, are hydrocarbons that feature one or more double bonds between two adjacent carbon atoms, which are important chemical raw materials [1]. Therein, light olefins such as C_2_H_4_ and C_3_H_6_ are widely used in the chemical synthesis of polymer materials, including fiber, rubber, and plastics, where the purity of monomers could decisively limit the corresponding product properties and application fields. Therefore, in order to meet the demand of downstream industries and generate high-purity olefins, it’s necessary to implement effective olefin/paraffin separation and remove the by-product and contaminants (e.g., alkane) [7]. Although various MOF membranes have already been reported for olefin/paraffin separation, the separation efficiency is still compromised due to similar physicochemical properties and molecular structures between olefins and alkanes, especially the approximate molecular size [119]. For example, the difference between the kinetic diameters of C_3_H_6_ (4.0 Å) and C_3_H_8_ (4.2 Å) is merely 0.2 Å, hence, the separation of olefin/paraffin is still full of challenges.

In C_2_H_4_/C_2_H_6_ separation, Chuah et al. [109] used HKUST-1 nanocrystalline as filler to prepare MMMs with ODPA-TMPDA and 6FDA-TMPDA matrixes, respectively. Due to the interaction between the unsaturated metal sites on UKUST-1 and the π electrons of ethylene, the C_2_H_4_ permeability of ODPA-TMPDA and 6FDA-TMPDA MMMs could achieve 16.0 ± 0.2 and 183 ± 3.8 Barrer, respectively. Compared to the corresponding pure polymeric membranes, the permeability of ODPA-TMPDA and 6FDA-TMPDA MMMs increased by 155% and 69%, respectively, while the selectivity of C_2_H_4_/C_2_H_6_ decreased slightly, thus making the separation performance of this MMM close to the upper bound. In addition to the polymeric matrix, various metal ions could also be used to prepare MMMs for C_2_H_4_/C_2_H_6_ separation. Chen et al. [110] prepared M-gallate(F)/6FDA-DAM MMM using M-gallate (M = Ni, Co, Mg) MOF fillers with the 6FDA-based PI matrix. The separation performance of M-galate(F)/6FDA-DAM MMM could achieve its best with a Ni-gallate(F) loading content of 20%. Meanwhile, C_2_H_4_ permeability reached 74.8 Barrer with C_2_H_4_/C_2_H_6_ selectivity of 2.55, which was 53.91% and 44.8% higher than that of 6FDA-DAM membranes, respectively.

For C_3_H_6_/C_3_H_8_ separation, Wang et al. [111] prepared the IOR-ZIF-8 MOF (IOR = inhibited Ostwald ripping) ultrathin film (thickness: ~180 nm) by adding PEG-NH_2_ inhibitor to inhibit the growth of ZIF-8 MOF using an IOR strategy, which improved the membrane separation performance of C_3_H_6_/C_3_H_8_. Among them, lone pair electrons on the amino group of PEG-NH_2_ and ether oxygen groups could coordinate with Zn^2+^ and compete with ligands, which reduced the surface free energy of small ions and inhibited the formation of large particles, greatly reducing the thickness of IOR-ZIF-8 MOF film. Note that the C_3_H_6_ permeability of the IOR-ZIF-8 MOF membrane could reach 386 GPU with a C_3_H_6_/C_3_H_8_ separation factor of 120. In addition, the thickness of the membrane can also be achieved by regulating the IOR process through the modification of the concentration, functional group, and molecular weight of the inhibitor. In addition to the preparation of ultrathin film, the reasonable selection of MOF and polymer matrix with similar properties could contribute to improving the separation performance of MMMs. Zhao et al. [112] synthesized PP-supported ZIF-8 membranes using ZIF-8 and polypropylene matrix through FCDS. Due to the slight difference in Young’s modulus between PP and ZIF-8, the PP-supported ZIF-8 membranes had superior flexibility, wherein the C_3_H_6_/C_3_H_8_ separation performance remained unchanged with the higher separation factor (i.e., 122 ± 13), even when the bending curvature is up to 92 m^−1^.

**Figure 7:**
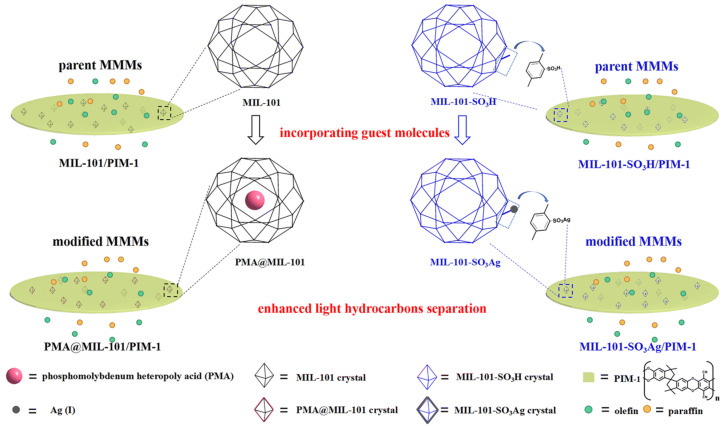
Schematic illustration of the preparation of PMA@MIL-101/PIM-1 and MIL-101-SO_3_Ag/PIM-1 MOF membranes for the light hydrocarbon separation. Reproduced with permission from [113], copyright 2022, Royal Society of Chemistry.

Note that some MOF-based membranes have been reported for simultaneous C_2_H_4_/C_2_H_6_ and C_3_H_6_/C_3_H_8_ separation with exceptional performance, in addition to the aforementioned sole function of C_2_H_4_/C_2_H_6_ or C_3_H_6_/C_3_H_8_ separation. In order to improve the light hydrocarbon separation performance of MMM with intrinsic microporosity formed by MIL-101(Cr) MOF and PIM-1 polymers, Feng et al. [113] prepared modified phosphomolybdenum heteropoly acid (PMA)@MIL-101/PIM-1 and MIL-101-SO_3_Ag/PIM-1 by loading PMA guest molecules into MIL-101(Cr), and the pore environment of MOF crystalline porous fillers was regulated by introducing PMA and Ag(I), which have a strong interaction with olefins (Figure 7). Compared to MIL-101/PIM-1 and MIL-101-SO_3_H/PIM-1, C_2_H_4_/C_2_H_6_ selectivity was increased to 2.88 (by 14%) and 3.47 (by 127%), respectively. Similarly, C_3_H_6_/C_3_H_8_ selectivity was increased to 5.96 (by 46%) and 3.89 (by 63%), respectively. Moreover, the C_2_H_4_/C_3_H_6_ permeance of modified PMA@MIL-101/PIM-1 and MIL-101-SO_3_Ag/PIM-1 MMM could reach up to 1632/1480 Barrers and 1456/1663 Barrers, respectively. Yang et al. [116] synthesized Zn_2_(bim)_4_ membranes through the in situ interfacial assembly (ISIA) strategy, which had a sieving effect on olefins due to flexible spacing derived from disorders and loose stacking between layers. Then, the modified Zn_2_(bim)_4_ membrane was obtained using the ionic liquid/Ag^+^ complex through a rational post-synthetic modification strategy, which has separation performance for both C_2_H_4_/C_2_H_6_ and C_3_H_6_/C_3_H_8_. Note that the permeabilities of C_2_H_4_ and C_3_H_6_ were 45.6 ± 23.7 GPU and 129.8 ± 40.4 GPU, respectively. Meanwhile, the corresponding separation factors were 12.0 ± 2.2 and 28.8 ± 3.8, respectively. Overall, this method endowed the inert membrane with olefin/paraffin separation performance.

## 4. Liquid Separation

Compared with gas separation, membrane separation technology is actually more prominent in the field of industrial liquid separation [120]. Commercial liquid separation processes include flocculation-sedimentation-filtration [121], adsorption [122], and membrane separation [123]. Among them, membrane separation is an energy-saving method, and polymer membranes have been applied for liquid separation. However, the low stability of polymer membranes compromises their separation performance. With the improvement of the liquid phase stability of MOF, MOF-based membranes could also be used for liquid phase separation in addition to gas phase separation [29,124], depending on molecular sieving mechanisms and chemical affinity differences. MOF-based membranes not only overcome the trade-off between permeability and selectivity of polymer membranes but also improve these two crucial parameters. Liquid phase separation applications of MOF-based membranes include water purification, organic solvent nanofiltration, and chiral separation (Table 2).

### 4.1. Water Purification

Approximately 780 million people in the world cannot guarantee access to clean water resources, and the shortage of fresh water is becoming a major threat to the development of human society. Therefore, water purification systems are very important, of which seawater desalination and wastewater recovery are considered potential for sustainable production of clean drinking water [135,136]. Membrane-based separations for water purification and desalination have been increasingly used to address the global challenges of water scarcity and pollution in aquatic environments, where MOF-based membranes are also widely used [137]. When MOF-based membranes are used for membrane distillation, the hydrophilicity, chemical stability, and thermal properties of the membranes could be further enhanced by the addition of MOF [138]. Moreover, Yang et al. [139] prepared PVDF nanofiber membranes loaded with 5 wt% MOF for membrane distillation for desalination, which displayed an ideal NaCl rejection rate of 99.99%. Furthermore, Zuo et al. [140] also used MOF-based membranes for membrane distillation and seawater desalination. Due to the addition of MOF, the hydrophobicity of the membrane is greatly enhanced, and the membrane prepared under optimum conditions achieved a good vacuum membrane distillation flux of 32.3 L m^−2^ h^−1^ at 60 °C. This study may open up a totally new approach for the design of next-generation high-performance membrane distillation membranes for seawater desalination.

The most difficult section of wastewater processing is water-soluble volatile organic compounds (VOCs), most of which are larger than water molecules in hydrodynamic diameter (*D*_h_). For instance, the *D*_h_ of *N*,*N*-dimethylformamide (DMF), aniline, dimethyl sulfoxide (DMSO), and *N*-methylpyrrolidone (NMP) molecules are 0.50 nm, 0.58 nm, 0.61 nm, and 0.69 nm, respectively [126]. Therefore, wastewater purification could be achieved using MOF membranes with the proper particle size, allowing water molecules to pass through while rejecting VOCs. When applied to the water treatment field, MOF-based membranes are often used in nanofiltration, ultrafiltration, forward osmosis, reverse osmosis, and so on [29].

#### 4.1.1. Reverse Osmosis and Forward Osmosis

A reverse osmosis (RO) membrane with a pore size less than 0.5 nm could be used to filter low molecular weight substances, such as salt ions and organic molecules [141]. RO refers to solvent molecules passing through from the high-pressure side of the membrane to the low-pressure side by applying a pressure greater than the osmotic pressure. RO has been widely used in desalination and water purification, producing nearly 65.5 million cubic meters per day (m^3^/day), accounting for 69% of the global desalination capacity [142]. Commonly used RO membranes, e.g., polyamide thin-film composite (TFC) membranes, could achieve high salt rejection (i.e., 99%) through size exclusion and charge repulsion. However, the removal efficiency of neutral contaminants in seawater, such as boron (~4–7 mg/L in seawater), is not high [143]. In order to improve the permeability and selectivity of TFC membranes, nanofillers (e.g., zeolites, carbon nanotubes, carbon molecular sieves, mesoporous silica, metal oxides, and MOFs) could be added to form thin-film nanocomposite (TFN) membranes [144]. However, the membrane selectivity of polyamide TFN could decline (e.g., low NaCl rejections of ≤50%) due to particle agglomeration and/or defects at the filler-polyamide interface that prompt the formation of non-selective voids. Therefore, in order to obtain TFN RO membranes with high separation performance, it is foremost important to improve the loading capacity of fillers and enhance interface compatibility with polyamide. Han et al. [126] carried out post-synthetic modification of MIL-101(Cr)-NH_2_ MOF filler nanoparticles to obtain MIL-101(Cr)–NH_2_–polyamide TFN membrane, which enhanced the interface compatibility between filler and polyamide, inhibited particle aggregation, and improved the separation performance of the membrane. The TFN membrane has a high rejection (i.e., 99.0–99.6%) of NaCl, MgCl_2_, Na_2_SO_4_, and MgSO_4_ with a water performance of 0.9 L m^−2^ h^−1^ bar^−1^ at 150 psi, which is 24.5% and 53.0% higher than that of the TFC membrane. Moreover, there are also excellent rejections for internal pollutants, where rejections for PEG200 and boric acid are 99.2% and 89.0%, respectively.

**Figure 8:**
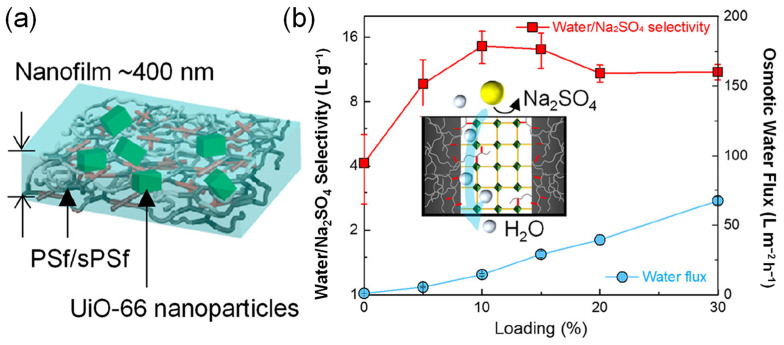
(**a**) Schematic illustration of self-standing MOF-based thin membranes (UiO66TFs). (**b**), Separation performance of the prepared UiO66TFs. Reproduced with permission from [126], copyright 2018, American Chemical Society.

Osmotic pressure-driven forward osmosis (FO) could cause solvent molecules in the waste-containing feed solution to enter the draw solution and intercept contamination in the feed solution [145], which is often used for seawater desalination and sewage treatment [29]. However, due to the internal concentration polarization of typical FO membranes, the water flux is usually far from the expected value [146]. The nano-composite membrane prepared by nano-modification with MOFs could reduce the transmission resistance and internal concentration polarization of the film. Liu et al. [126] prepared self-standing MOF-based thin films (UiO66TFs) with nano-sizing UiO-66, sulfonated PSF additives, and PSF through a facile solution casting strategy, which is used for water purification based on forward osmosis (Figure 8). Herein, the sulfonated PSF in UiO66TFs could form an inter/intra-molecular hydrogen bond with the PSF matrix, causing the MOF and the polymer matrix to have high dispersion and interface bonding. Meanwhile, the pore size of UiO66TFs is between water molecules and hydrogenated ions, thus separating the hydrogenated ions. Moreover, the membrane could eliminate ICP and increase water flux, depending on its supportive-free, symmetric structure. Therefore, UiO66TFs have higher separation performance than polymer membranes, which could improve the ion selectivity and water flux by 56 times (i.e., 1.41 L m^−2^ h^−1^ bar^−1^), with water/Na_2_SO_4_ permselectivity increasing by 3 fold (i.e., 13.5 L g^−1^). Moreover, the Na_2_SO_4_ rejection was improved to 94–96% as well.

#### 4.1.2. Nanofiltration

Nanofiltration (NF) is a membrane separation method driven by low pressure, of which the selectivity depends largely on pore size distribution and Donnan effects [147]. The filtration capacity for ions or molecules is between ultrafiltration and reverse osmosis due to the characteristic pore size (i.e., 0.5–2 nm) and cut-off molecular weight (MWCO) (i.e., 100–2000 Da). However, given its charged surface in water [148], nanofiltration has a higher selectivity for ions (e.g., high divalent ion rejection and low monovalent ion rejection) compared to ultrafiltration and reverse osmosis [148]. Hence, improving the separation performance of nanofiltration membranes has aroused important research interests. Nanofiltration membranes are often used for desalination and dye removal. For example, Cong et al. [127] prepared polycrystalline MOF-303 membranes for water desalination using water-soluble MOF-303 (Al(OH)(HPDC); HPDC = 1H-pyrazole-3,5-dicarbox-ylate) and α-Al_2_O_3_ substrates by the in situ hydraulic synthesis method. Considering that MOF-303 has one-dimensional (1D) rhombic channels (open space of ~0.6 nm) along the α Axis, and the pore size being larger than the kinetic diameter of water molecules (i.e., 0.28 nm) but smaller than the kinetic diameter of common hydrogenated ions (i.e., ≥0.66 nm), which enables MOF-303 membranes to conduct water softening through a size-exclusion mechanism, with high rejection for divalent ions such as MgCl_2_ (i.e., 93.5%) and Na_2_SO_4_ (i.e., 96.0%). In addition, the membrane has an unprecedented water permeability of 3.0 L·m^−2^ h^−1^ bar^−1^·μm. Meanwhile, it is worth noting that the membrane features excellent stability and low material costs, making it a promising industrial product with practical applications. Xiao et al. [128] prepared a TA-Zn^2+^ based network by LbL self-assembly method with tannic acid (TA) and Zn^2+^, which was then used as a precursor to prepare a ZIF-8/(TA-Zn^2+^)_2_/PES nanofiltration membrane for effective desalination. The separation performance was improved due to the fact that the LbL process provided sufficient controllability to prepare a continuous ultra-thin ZIF-8 layer, of which pure water permeance reached 5.1 L m^−2^ h^−1^ bar^−1^. Meanwhile, the rejections of NaCl and Na_2_SO_4_ were 55.2% and 93.6%, respectively. However, Meng et al. [129] prepared a BUT-8 (A)/PEI-HPAN-50 (HPAN = hydrolyzed polyacrylonitrile) mixed matrix nanofiltration membrane using a BUT-8(A) (BUT-8(A) = Cr_3_(μ_3_-O) (H_2_O)_3_(NDC(SO_3_H_5/6_)_2_)_3_, NDC(SO_3_H)_2_^2−^ = 4,8-disulfonaphthalene-2,6-dicarboxylate) and PEI matrix, which could be used for dye removal in sewage. Therefore, the UT-8(A)/PEI-HPAN-50 membrane exhibited a high water permeance for aqueous dye solutions of methyl blue, congo red, acid fuchsin, and methyl orange (i.e., 396, 683, 490, and 510 L m^−2^ h^−1^ MPa^−1^, respectively), while the corresponding dye rejections were 98.3%, 99.8%, 89.3%, and 82.1%, respectively.

MOF can not only be used for desalination to achieve the rejection of MgCl_2_ and Na_2_SO_4_, but can also separate lithium ions (Li^+^) from water to alleviate the dilemma of lithium-ion demand brought by booming new energy sources such as electric vehicles [149,150]. For example, Guo et al. [151] prepared PSS@HKUST-1-6.7 MMMs using polystyrene sulfonate and HKUST-1 MOF. The as-prepared product exhibited good separation performance for Li+, with ideal separation factors for Li^+^/Na^+^ (i.e., 35), Li^+^/K^+^ (i.e., 67), and Li^+^/Mg^2+^ (i.e., 1815), respectively. Liang et al. [152] prepared the SSP@ZIF-8-10% membrane by loading sulfonated spiropyran (SSP) onto ZIF-8 crystals. The as-prepared membrane has ideal selectivities of 77, 112, and 4913 for Li^+^/Na^+^, Li^+^/K^+^, and Li^+^/Mg^2+^ in the dark at 25 °C, respectively.

#### 4.1.3. Ultrafiltration

Ultrafiltration (UF) membranes feature excellent stability, high separation efficiency, low operation pressure, and low operating temperature, which could effectively separate high molecular weight substances (such as proteins), suspended nanoparticles, and bacteria from sewage, and of which the aperture is 2 nm–0.1 μm [153]. It is well known that the high-efficiency UF process must have high flux, a high rejection rate, and prominent antifouling properties, which are mainly affected by the pore structure and surface properties of the UF membrane. Therefore, in order to improve membrane performance, hydrophilic inorganic materials, such as TiO_2_, SiO_2_, ZnO, graphene oxide (GO), and their corresponding nanocomposites, have been widely incorporated into polymeric UF membranes to tailor the porous structure and other fundamental properties of membranes [154]. However, there exist dilemmas such as nanoparticle polymerization and poor compatibility with the polymer that compromise the separation performance of UF. Therefore, compared with traditional inorganic materials, MOFs have better compatibility with polymer matrix due to the existence of organic ligands, which could be combined in the UF membrane to improve the separation performance. Sun et al. [130] synthesized hydrophilic hollow zeolitic imidazolate framework-8 (hZIF-8) through a meticulous surface functionalization-assisted etching approach using tannic acid (TA) and PSF casting solutions to fabricate novel hybrid UF membranes (PSF/hZIF hybrid UF membranes). As an etching agent, TA not only endowed hZIF-8 with a unique hollow structure and highly hydrophilic surface but also retained the intrinsic frameworks, enabling the facile design and fabrication of high-performance UF membranes for water processing. Considering the well-tailored surface properties and nanostructure of hZIF-8, the obtained PSF/hZIF hybrid UF membranes presented superiorly enhanced water permeation (i.e., 597 L m^−2^ h^−1^), which was 2.8-fold of the pristine PSF membrane (i.e., 210 L m^−2^ h^−1^), while well maintaining the rejection property. Yang et al. [131] prepared excellent cellulose acetate (CA) ultrafiltration membranes, i.e., CA/MOF@GO membranes, using CA and HKUST-1@GO composites as fillers. Therein, HKUST-1 was selected as the GO modifier. Compared with CA/GO membranes, the CA/MOF@GO membrane has higher hydrophilicity with a water permeate flux of 183.51 L m^−2^ h^−1^ and could prevent the stacking of GO layers, which possess a flux recovery ratio of 88.13% and a total fouling ratio of 40.32%.

Notably, MOF can not only be used to prepare UF, thereby achieving water purification, but also has an adsorption effect on pharmaceutical waste in water [155], forming a hybrid system with a UF membrane for separating pharmaceutical waste from water. For example, Kim et al. [156] combined MIL-101(Cr) with commercial UF to form the MOF-UF hybrid system, where MOF can not only remove selected pharmaceutical active compounds (PhACs) and natural organic matter (NOM), but also reduce fouling in adsorbent-UF hybrid systems. The average retention rates of MOF-UF hybrid systems for PhACs and NOMs were 53.2% and 86.1%, respectively, higher than those of UF alone (i.e., PhACs: 36.7%; NOMs: 75.7%) and powered activated carbon UF systems (i.e., PhACs: 48.6%; NOMs: 79.2%). Moreover, Dai et al. [157] grafted ethylenediamine to MIL-101(Cr) to obtain dually charged ED-MIL-101(Cr). MOF-TFN was then prepared using dually charged MOF as prepared, which presented a great separation effect on both negatively (e.g., ketoprofen, diclofenac, bezafibrate) and positively (e.g., terbutaline, atenolol, fluoxetine) charged PhACs, with rejection rates of up to 90%.

#### 4.1.4. Pervaporation

Pervaporation is a process that can achieve separations of liquid mixtures by selective permeation and vaporization through a membrane, which could be used as an alternative separation method for some liquid mixtures not suitable for filtration and separation [158]. Through pervaporation, MOF-based membranes could execute the separation of organic liquids for dehydration and organic mixtures [159]. However, the MOF-based membrane could also purify water through pervasion. Peng et al. [132] prepared a DM-ZIF-8-m photothermal membrane from modified-ZIF-8 and polyaniline (PANI) for wastewater purification containing water-soluble VOCs. Therein, PANI worked as a photothermal layer and converted solar energy into heat energy with the water vapor generated. Meanwhile, the pore size of modified ZIF-8 is 0.34 nm, which is between the size of water molecules and most VOCs, thus possessing the screening function of removing VOCs from water. Therefore, the DM-ZIF-8-m membrane had a high photothermal conversion efficiency and a molecular sieving effect. Under 1 sum radiation (1 Kw m^−2^), the evaporation rate of water could reach the ideal value of 1.0 kg m^−2^ h^−1^, with a 99% rejection rate of VOCs. Moreover, the rejection rate of VOCs could still reach 99% even when the VOC content of water is as high as 400 mg/L. This method not only realized the purification of water with high VOC content but also exploited sustainable energy, which is of great significance to protect the environment and cope with climate change.

### 4.2. Organic Solvent Nanofiltration

The organic solvent nanofiltration (OSN) membrane has remarkable stability and high permeability in a wide range of organic solvents with different polarities, of which the pore size is below 2 nm. The OSN membrane could separate valuable small active molecules with MWs of 200–1000 Da from organic solvents, which are often used for filtration and concentration of organic solutions [160]. Unlike acute aqueous nanofiltration, the separation performance of OSN is based solely on the physical size exclusion effect rather than electrical interaction [161]. The OSN membrane has a relatively loose structure with large pores and a high solvent flux. However, the selectivity of the OSN membrane is relatively low to some extent. Herein, the tunable porosity and pore size of MOFs can create selective cavities and paths to increase solvent flux and maintain a high rejection that could be achieved by adding MOF filler to the OSN membrane [162].

**Figure 9:**
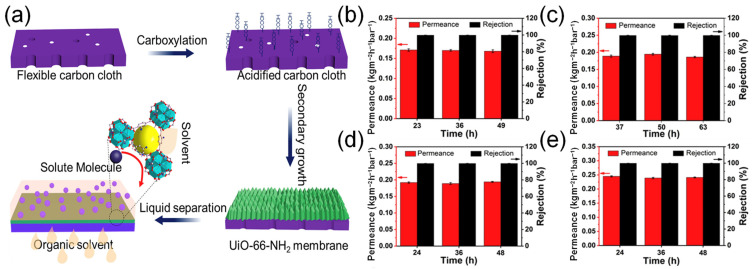
(**a**) Schematic illustration of the preparation processes of polycrystalline UiO-66-NH_2_. (**b**–**e**) Separation performance of the prepared UiO-66(Zr)–NH_2_ membrane for the removal of MB (in dichloromethane), OR (in dichloromethane), NR (in dichloromethane), and NR (in methanol), respectively. Reproduced with permission from [134], copyright 2020, Elsevier.

Various strategies have been established to modify established membrane configurations for OSN, such as ceramic membranes, TFC membranes, and integrally skinned asymmetric (ISA) membranes. Specifically, the TFC membrane is a representative MOF-based membrane for organic solvent nanofiltration. Chen et al. [133] prepared a TFC nanofiltration membrane with high organic solvent flux by in situ growth of a HKUST-1 MOF intermediate layer on a crosslinked PI matrix, wherein a thin and dense PI separation layer with excellent separation performance was formed between the highly porous HKUST-1 layers. The membrane realized an eminent methanol permutation flux up to 9.59 L m^−2^ h^−1^ bar^−1^, while maintaining the rejection of the negatively charged Brilliant Blue G 250 dye (MW = 858.05 g mol^−1^) at 98.8%. Moreover, the TFC membrane demonstrates satisfactory stability in organic solvents such as DMF, suggesting the potential for OSN applications in chemical industries. In addition to the TFC membrane, the polycrystalline MOF membrane has also demonstrated well-pleasing separation performance and solvent resistance in the field of organic solvent nanofiltration. Cai et al. [134] prepared a polycrystalline UiO-66(Zr)–NH_2_ membrane through in situ solvothermal synthesis using flexible carbon cloth as a substrate (Figure 9). The membranes possess submicron thicknesses with a certain flexibility and an angular bending tolerance of 10°, which exhibited great separation performance in OSN dependent on their excellent solvent resistance. Meanwhile, membrane rejections of oil red O, Nile red (NR), and methylene blue (NB) in dichloromethane (DCM) solutions were calculated to be 99.95, 99.85, and 99.90%, respectively, with a high flux for dichloromethane (ca. 0.17 kg m^−2^ h^−1^ bar^−1^).

### 4.3. Chiral Resolution

Due to significant differences in biological activity and pharmacological properties between isomers of chiral compounds (e.g., cis-[Pt(NH_3_)_2_Cl_2_] is an anticancer drug but trans-[Pt(NH_3_)_2_Cl_2_] is a toxin), chiral resolution (i.e., enantiomer separation) has attracted widespread attention [163]. Conventional chiral resolution techniques include crystallization, kinetic resolution, chromatography, and membrane-based separation. Considering that MOF has a high specific surface area, adjustable pore sizes, and variable chemical functionalities, the homochiral MOF-based membrane is promising for chiral enantiomer separation. Preparation methods for homochiral MOF-based membranes include chiral linkage, postsynthetic modification, and self-assembly driven by the chiral environment [164]. Since enantiomers have different stereo configurations, their interaction with the ligand of MOF might be different (e.g., hydrogen bonding, π-π stacking, van der Waals forces, etc.) [165]; therefore, MOF-based membranes could separate enantiomers through chiral recognition and simple molecular sieving.

**Figure 10:**
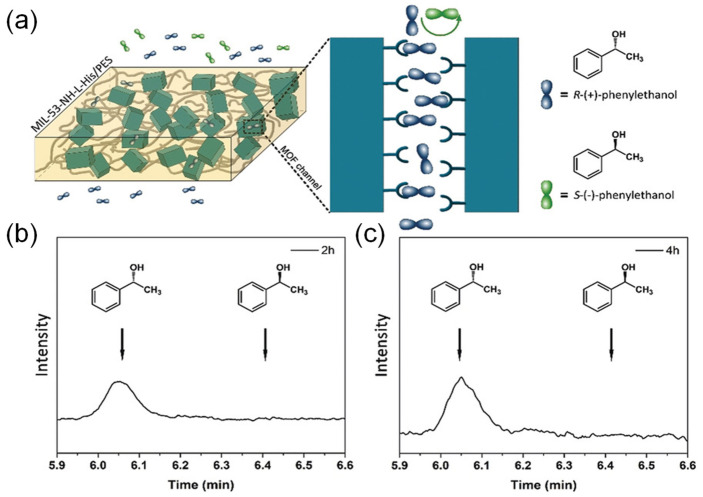
(**a**) Schematic illustration of the selective transport of *R*-(+)- and *S*-(−)-1-phenylethanol through the MIL-53-NH-l-His channel. Gas chromatograms of resolved 1-phenylethanol enantiomers after separation for (**b**) 2 h and (**c**) 4 h. Reproduced with permission from [164], copyright 2019, John Wiley and Sons.

Lu et al. [164] prepared MIL-53-NH-_L_-His and MIL-53-NH-l-Glu nanocrystals by post-synthetic modification of achiral MIL-53-NH_2_ MOF using l-histidine (l-His) and l-glutamic acid (l-Glu), respectively. The intermediates were then combined with PES matrix to form homochiral MMMs, which exhibited excellent enantioselectivity for racemic 1-phenylethanol with the highest enantiomeric excess value of 100% (Figure 10). Note that the natural amino acid can not only transform the achiral MIL-53-NH_2_ into the homochiral MOF but is also applicable to ZIF. The homochiral l-His-ZIF-8 membrane reported by Chan et al. [166] exhibited good selectivity for the *R*-enantiomer of 1-phenylethanol over the *S*-enantiomer, showing a high enantiomeric excess value of 76%. Although homochiral MOF-based membranes displayed effective chiral resolution, membrane selectivity for enantiomers could decrease with permeation time. In this context, Lu et al. [167] prepared CD-MOF/PES MMM using cyclodextrin (CD)-MOF as chiral selector and PES matrix. The membrane revealed nearly 100% enantioselectivity of *R*-(+)-1-phenylethanol over *S*-(-)-1-phenylethanol when non-polar n-hexane is used as a solvent.

In addition to being used as membranes for chiral separation, MOF can also be used in capillary electrochromatography (CEC) for chiral drug separation based on electroosmotic flow [168]. Ding et al. [169] prepared a pepsin-ZIF-8 poly-(GMA-*co*-EDMA) (poly-(GMA-*co*-EDMA = poly(glycidyl methacrylate)-*co*-(ethylene dimethacrylate))column by mixing ZIF-8 into poly-(GMA-*co*-EDMA) monoliths in CEC and then covalently attaching the chiral selector (i.e., pepsin) to the surface of the amino-modified ZIF-8 by the Schiff base method. Note that the separation resolution of six chiral drugs (i.e., chloroquine (CHQ), hydroxychloroquine (HCQ), nefopam (NEF), clenbuterol (CLE), amlodipine (AML), and hydroxyzine (HXY)) using the pepsin-ZIF-8-poly(GMA-*co*-EDMA) column demonstrated remarkable improvement in the resolution (i.e., CHQ: 0.45→1.97; HCQ: 0.34→2.50; NEF: 0.27→0.81; CLE: 0→0.81; AML: 0.1→0.72; HXY: 0.39→1.43). Similarly, Sun et al. [170] used lipase immobilized MIL-100 (Fe) biocomposites as a chiral stationary phase for capillary electrochromatographic enantioseparation. The performance of the porous column was evaluated by enantioseparating amino acid enantiomers with a high resolution of over 2.0. Moreover, the enantio-resolutions of phenylephrine, phenylsuccinic acid, chloroquine, and zopiclone were also greater than 2.0.

## 5. Future Perspectives

MOF-based membranes are competitive candidates for membrane separation and have attracted extensive attention. However, traditional approaches to MMMs, such as designing new MOFs or challenging the matching of multiple fillers and polymer matrices, make it difficult to fully meet different separation requirements. Notably, defect engineering could make the same MOF-based membrane meet different separation requirements by simply regulating the concentration of defects in the MOF-based membrane. Traditional approaches for preparing high performance pure MOF membranes include grain boundary structure regulation, however, which is unavoidable for polycrystalline MOF membranes. However, for MOF glass hollow fiber membranes, it is expected to eliminate grain boundaries in polycrystalline MOF membranes. Furthermore, we could pay more attention to the fabrication of novel MOF glass hollow fiber membranes for their facile processability and high thermal stability for large-scale applications.

Notably, MOF-in-COF membranes could be a breakthrough, benefiting from the synergy of precise size sieving and fast molecular transport through MOF-in-COF channels. Meanwhile, numerous combinations of MOFs and COFs in robust MOF-in-COF membranes demonstrate the versatility and promising prospects for membrane separation of gas, liquid, etc. However, the functional stimuli-responsive moieties of our previous results pose another potential for novel MOF-based membranes with precise pore sizes triggered by stimulated responsibility, probably contributing to better membrane permeance and selectivity.

## 6. Conclusions

In this review, we briefly summarized the recent achievements of MOF membranes (pure MOF membranes and MMMs) for separation and discussed the separation performance and relevant influencing factors, including framework flexibility, defect and grain orientation of pure MOF membranes, MOF filler aggregation, plasticization and aging of polymer matrix, and interfacial compatibility of MMMs. Meanwhile, MOF membrane applications for gas are described in detail, such as CO_2_, H_2_, and olefin/alkane separation. Furthermore, MOF-based membranes for liquid phase separation are comprehensively reviewed, including water purification, organic solvent separation, and chiral resolution. Overall, we provide broad insight into this important area of great concern and the prospects for the future of MOF-based membranes as promising functional materials.

## Figures and Tables

**Table 1:** Summary of gas separation performance of MOF membranes discussed in this review.

MOF Membrane	Substrate	Permeance (Barrer)	Selectivity Factor	Ref.
MIL-160/CAU-10-F	Al_2_O_3_	CO_2_: 2148	CO_2_/CH_4_: 78	[100]
PI-IL/MOF	PI	CO_2_: 7.16	CO_2_/CH_4_: 95.1	[101]
C-Z8/P-5	Pebax	CO_2_: 286	CO_2_/N_2_: 137	[102]
UiO-66-NH_2_-PVA-PVAm	Poly(vinyl amine)	CO_2_: 75	CO_2_/N_2_: 45	[103]
UiO-66/PEGDA	Poly(ethylene glycol) diacrylate	CO_2_: 470	CO_2_/N_2_: 41	[104]
ZIF-8/PIM-1	PIM-1	CO_2_: 6338	CO_2_/N_2_: 24.4 ± 2.3	[60]
CAU-10-H	α-Al_2_O_3_	CO_2_: 500	CO_2_/N_2_: 42 CO_2_/CH_4_: 95	[105]
Zn_2_(Bim)_4_ SSCM	PVDF	H_2_: 315 ± 22	H_2_/CO_2_: 1084 ± 80	[106]
ZIF-8/GNR/alumina	alumina	H_2_: 8449	H_2_/CO_2_: 142	[49]
ZIF-8/PI-CuBTC	Cu net	H_2_: 39,305	H_2_/CH_4_: 71	[55]
Branched-ZIF-8	6FDA-DAM	H_2_: 1528	H_2_/CH_4_: 19.45	[107]
*c*-oriented ZIF-95	α-Al_2_O_3_	H_2_: 1540	H_2_/CO_2_: 32.2 H_2_/CH_4_: 53.7	[57]
ZIF-9	α-Al_2_O_3_	H_2_: 1469	H_2_/CO_2_: 21.5 H_2_/CH_4_: 8.2 H_2_/N_2_: 14.7	[54]
HPM	PVDF	H_2_: 17,600	H_2_/CO_2_: 361 H_2_/CH_4_: 541 H_2_/N_2_: 482	[108]
ZIF-7_22_-8	Anodic aluminum oxide	CO_2_: 24.37 H_2_: 44.68	CO_2_/CH_4_: 25 CO_2_/N_2_: 20 H_2_/CH_4_: 71	[50]
MIL-125-TG	α-Al_2_O_3_	CO_2_: 500 H_2_: 3548	CO_2_/N_2_: 38.7 H_2_/N_2_: 64.9 H_2_/CH_4_: 40.7	[56]
UTSA-280/6FDA-polyimide	6FDA-DAM:DABA (3:2) polymer matrix	C_2_H_4_: 6.49	C_2_H_4_/C_2_H_6_: 4.94	[66]
801@Ni74(26)durene	UiO-66-NH_2_	C_2_H_4_: 26	C_2_H_4_/C_2_H_6_: 5.91	[68]
HKUST-1/ODPA-TMPDA	ODPA-TMPDA	C_2_H_4_: 16.0 ± 0.2	C_2_H_4_/C_2_H_6_: 2.5 ± 0.5	[109]
HKUST-1/6FDA-TMPDA	6FDA-TMPDA	C_2_H_4_: 183 ± 3.8	C_2_H_4_/C_2_H_6_: 2.4 ± 0.1	[109]
M-gallate(F)/6FDA-DAM	6FDA-DAM	C_2_H_4_: 74.8	C_2_H_4_/C_2_H_6_: 2.55	[110]
Zn_82_Co_18_-ZIF	ZIF-67	C_3_H_6_: 31	C_3_H_6_/C_3_H_8_: 200	[58]
Ni-MOF/PDA/6FDA-DAM	6FDA-DAM	C_3_H_6_: 90	C_3_H_6_/C_3_H_8_: 75	[67]
UiO-66/6FDA-DAM	6FDA-DAM polyimide	C_3_H_6_: 237	C_3_H_6_/C_3_H_8_: 9.8	[71]
IOR-ZIF-8	Anodic aluminum oxide	C_3_H_6_: 69.5 ± 2.2	C_3_H_6_/C_3_H_8_: 120 ± 10	[111]
PP-supported ZIF-8	Polypropylene	C_3_H_6_: 17.7 ± 7.5	C_3_H_6_/C_3_H_8_: 122 ± 13	[112]
PMA@MIL-101/PIM-1	PIM-1	C_2_H_4_: 1632 C_3_H_6_: 1480	C_2_H_4_/C_2_H_6_: 2.88 C_3_H_6_/C_3_H_8_: 5.96	[113]
MIL-101-SO_3_Ag/PIM-1	PIM-1	C_2_H_4_: 1456 C_3_H_6_: 1663	C_2_H_4_/C_2_H_6_: 3.47 C_3_H_6_/C_3_H_8_: 3.89	[113]
Zn_2_(bim)_4_	Al_2_O_3_	C_2_H_4_: 9.12 ± 4.7 C_3_H_6_: 26.0 ± 8.1	C_2_H_4_/C_2_H_6_: 12.0 ± 2.2 C_3_H_6_/C_3_H_8_: 28.8 ± 3.8	[114]

**Table 2:** Summary of liquid separation performance of MOF membranes discussed in this review.

MOF Membrane	Substrate	Permeance (L m^−2^ h^−1^ bar^−1^)	Rejection Rate (%)	Ref.
MIL-101(Cr)–NH_2_–polyamide	Polyamide	water: 0.9	NaCl, MgCl_2_, Na_2_SO_4_, and MgSO_4_: 99–99.6 PEG200: 99.2 Boric acid: 89.0	[125]
UiO66TFs	PSF	water: 1.41	Na_2_SO_4_: 94–96	[126]
MOF-303	α-Al_2_O_3_	water: 0.75	MgCl_2_: 93.5 Na_2_SO_4_: 96.0	[127]
ZIF-8/(TA-Zn^2+^)_2_/PES	PES	water: 5.1	NaCl: 55.2 Na_2_SO_4_: 93.6	[128]
BUT-8 (A)/PEI-HPAN-50	HPAN	Water: 39.6	methyl blue: 98.3	[129]
		Water: 68.3	congo red: 99.8	
		Water: 49	acid fuchsin: 89.3	
		Water: 51	methyl orange: 82.1	
PSF/hZIF	PSF	water: 298.5	BSA: >98	[130]
CA/MOF@GO	GO	water: 122.3	BSA: 95.4	[131]
DM-ZIF-8-m	Polyaniline	water: 1.0	VOCs: 99	[132]
HKUST-1/PI	PI	Methanol: 9.59	Brilliant Blue G 250: 98.8	[133]
UiO-66(Zr)–NH_2_	Carbon cloth	Dichloromethane: 0.13	oil red O: 99.95 NR: 99.85 NB: 99.90	[134]

## Data Availability

Not applicable.
